# Aerosolization inhalation of non-typeable *Haemophilus influenzae* outer membrane vesicles contributing to neutrophilic asthma

**DOI:** 10.3389/fmicb.2023.1226633

**Published:** 2023-07-26

**Authors:** Ying Zhang, Hongbo Wang, Yanqiu Zhang, Peiliang Zhao, Yanan Li

**Affiliations:** Department of Pediatric Respiratory, The First Hospital of Jilin University, Changchun, China

**Keywords:** neutrophilic asthma, non-typeable *Haemophilus influenzae*, outer membrane vesicles, IL-1β, IL-17

## Abstract

**Background:**

Neutrophilic asthma is poorly responsive to corticosteroids, and the mechanism underlying its pathogenesis remains unclear. Non-typeable *Haemophilus influenzae* (NTHi) is the most common bacterium found in induced sputum from patients with neutrophilic asthma. NTHi can release outer membrane vesicles (OMVs), which transfer biomolecules to host cells and the external environment. However, the role and mechanisms of NTHi OMVs in the pathogenesis of neutrophilic asthma remain unclear.

**Methods:**

We conducted assays to investigate whether NTHi OMVs can induce neutrophilic asthma when inhaled. We isolated and purified NTHi OMVs and administered them via a nebulizer to ovalbumin (OVA)-sensitized mice. We collected and sequenced serum, blood, bronchoalveolar lavage fluid, and lung tissue from each group and gathered lung function data.

**Results:**

Inhaled NTHi OMVs-induced neutrophilic asthma in OVA-sensitized mice. High-throughput sequencing revealed that NTHi OMV inhalation in OVA-sensitized mice significantly enriched inflammatory and immune-related signaling pathways. We found increased transcription and secretion of interleukin (IL)-1β and IL-17, which may contribute to neutrophilic asthma. Furthermore, we discovered that airway epithelium is the first receptor cell of NTHi OMVs and releases IL-1β. These findings suggest that NTHi OMVs could be a potential target for neutrophilic asthma therapy.

## Highlights

- Outer membrane vesicles (OMVs) of non-typeable *Haemophilus influenzae* (NTHi) can enter the airway epithelium cells of mice by aerosolization inhalation.
- NTHi OMVs could induce neutrophilic inflammation in ovalbumin (OVA)-sensitized mice.
- NTHi OMVs combined with OVA-sensitized mice were significantly enriched in interleukin (IL)-1β inflammatory and IL-17 immune-related signaling pathways.

## Introduction

Asthma is a respiratory condition characterized by a history of symptoms such as wheezing, shortness of breath, chest tightness, and coughing. It is a complex and heterogeneous disease of the airways (Zhang et al., 2020). The primary feature of asthma is a chronic inflammation of the airways (Hammad and Lambrecht, 2021). Based on the relative numbers of immune cells in sputum, asthma can be classified as eosinophilic asthma, neutrophilic asthma, mixed granulocytic asthma, or paucigranulocytic asthma (Pavord et al., 1999). The Global Initiative for Asthma (GINA) guidelines are widely used worldwide for asthma management and prevention strategies (Chipps et al., 2022). According to GINA, the main prescriptions for asthma cases are inhaled corticosteroids (ICS) and beta-2 receptor agonists. It is well-known that eosinophilic airway inflammation responds well to ICS, but non-eosinophilic subtypes, particularly neutrophilic asthma, respond poorly to corticosteroids (Pavord et al., 1999). The mechanism of neutrophilic asthma remains unclear.

With the advancement of microbial identification technologies, studies have confirmed that microbes’ diversity and community composition are related to chronic airway inflammation in asthma (Bisgaard et al., 2007; Zhang et al., 2020; Ackland et al., 2022). Furthermore, the colonization of *Haemophilus influenzae*, *Moraxella catarrhalis*, and *Streptococcus pneumoniae* in newborns correlates with the morbidity rate of asthma (Gomez and Prince, 2008; Taylor et al., 2018). Non-typeable *Haemophilus influenzae* (NTHi) is the most common bacterium found in the sputum of patients with neutrophilic asthma and can be detected during both stable and exacerbation periods of asthma (Wood et al., 2010; Iikura et al., 2015; Zhang et al., 2020). NTHi infection destroys the airway epithelial barrier *in vitro* through decreased expression of the tight-junction protein E-cadherin (Kaufhold et al., 2017). Furthermore, NTHi infection stimulates peripheral blood mononuclear cells and alveolar macrophages (Ackland et al., 2022). Although NTHi is an intracellular pathogen that targets and settles in macrophages and airway epithelial cells for a prolonged period, in one study NTHi promoted asthma progression for 16 days after the infection had cleared (Essilfie et al., 2011). The mechanism by which NTHi infection modulates the airway immune response and chronic inflammation, particularly in neutrophilic asthma, is not well-understood (Clementi and Murphy, 2011).

Outer membrane vesicles (OMVs) are spherical lipid bilayers released by bacterial membrane vesicles (MVs), with sizes ranging from 20 to 400 nm in diameter, that affect various biological processes, including virulence, horizontal gene transfer, export of cellular metabolites, phage infection, and cell-to-cell communication (Brown et al., 2015; Schwechheimer and Kuehn, 2015; Orench-Rivera and Kuehn, 2016). Evidence suggests that OMVs deliver lipopolysaccharide (LPS) from Gram-negative bacteria into the cytosol, triggering a caspase-11-dependent response *in vitro* and *in vivo* (Vanaja et al., 2016). Furthermore, OMVs produced by NTHi transport nucleic acids, proteins, lipopolysaccharide, and phospholipids to regulate bacterial stress response and modulate host immune responses in respiratory epithelial cells (Sharpe et al., 2011). NTHi OMVs elicit upregulation of interleukin (IL)-8 in host epithelial cells through pathogen-associated molecular patterns (PAMPs). However, the role and mechanisms by which NTHi OMVs contribute to the pathogenesis of neutrophilic asthma have yet to be developed.

In this study, we aimed to monitor NTHi-released OMVs inhalation to induce neutrophilic asthma and confirm the roles of NTHi OMVs in neutrophilic pathogenesis.

## Results

### Inhalation of NTHi OMVs increased the mice’s airway hyper-responsiveness

**Figure 1:**
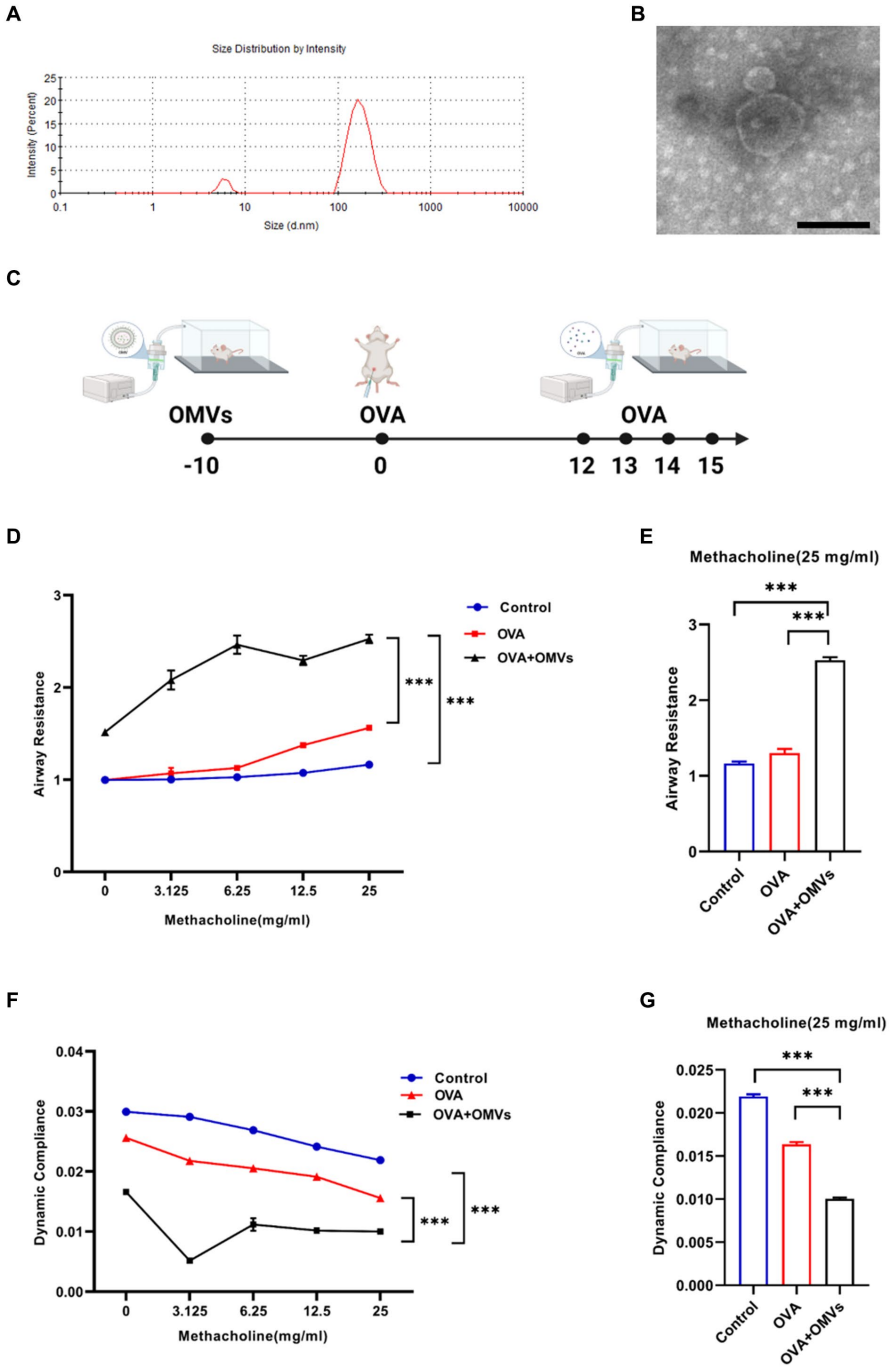
Inhalation of NTHi OMVs increased the mice’s airway hyper-responsiveness. **(A)** The diameter of NTHi OMVs detected by NanoSight. **(B)** Representative image of NTHi OMVs identified by TEM. Scale bar: 100 nm. **(C)** The experimental schedule for aerosol inhalation of NTHi OMVs in OVA-sensitized mice. **(D)** The airway resistance responding to different doses of acetyl-β-methylcholine chloride. Were obtained from control, OVA and OVA+OMVs induced groups by DSI Buxco RC FinePointe system. Each group contained 5 mice. **(E)** The airway resistance responding to 25 mg/mL acetyl-β-methylcholine chloride. Were examined by control, OVA and OVA+ OMVs induced groups using DSI Buxco RC FinePointe system. Each group contained 5 mice. **(F)** The dynamic lung compliance in response to increasing concentrations of acetyl-β-methylcholine chloride. Were obtained from control, OVA and OVA+OMVs induced groups by DSI Buxco RC FinePointe system. Each group contained 5 mice. **(G)** The dynamic lung compliance in response to 25 mg/mL acetyl-β-methylcholine chloride. Were examined by control, OVA and OVA+ OMVs induced groups using DSI Buxco RC FinePointe system. Each group contained 5 mice.

To characterize OMVs released from NTHi, we purified NTHi OMVs from cell-free culture supernatants using a detergent-free ultracentrifugation method. NanoSight analysis showed that the diameter of NTHi OMVs ranged from 100 to 300 nm (Figure 1A). TEM analysis revealed that the morphology of NTHi OMVs was nearly spherical (Figure 1B).

To investigate the regulatory effect of NTHi OMVs on the development and severity of asthma, we induced mice with OVA+NTHi OMVs according to the experimental schedule shown in Figure 1C. We then examined the mice’s lung function to assess airway hyper-responsiveness (AHR). Our results showed that inhalation of NTHi OMVs upregulated the mice’s airway resistance (Figures 1D,E) and reduced lung dynamic compliance (Figures 1F,G). These findings suggest that NTHi OMVs inhalation can increase mice’s OVA-induced AHR and airway obstruction.

### NTHi OMVs inhalation induced inflammation infiltration

**Figure 2:**
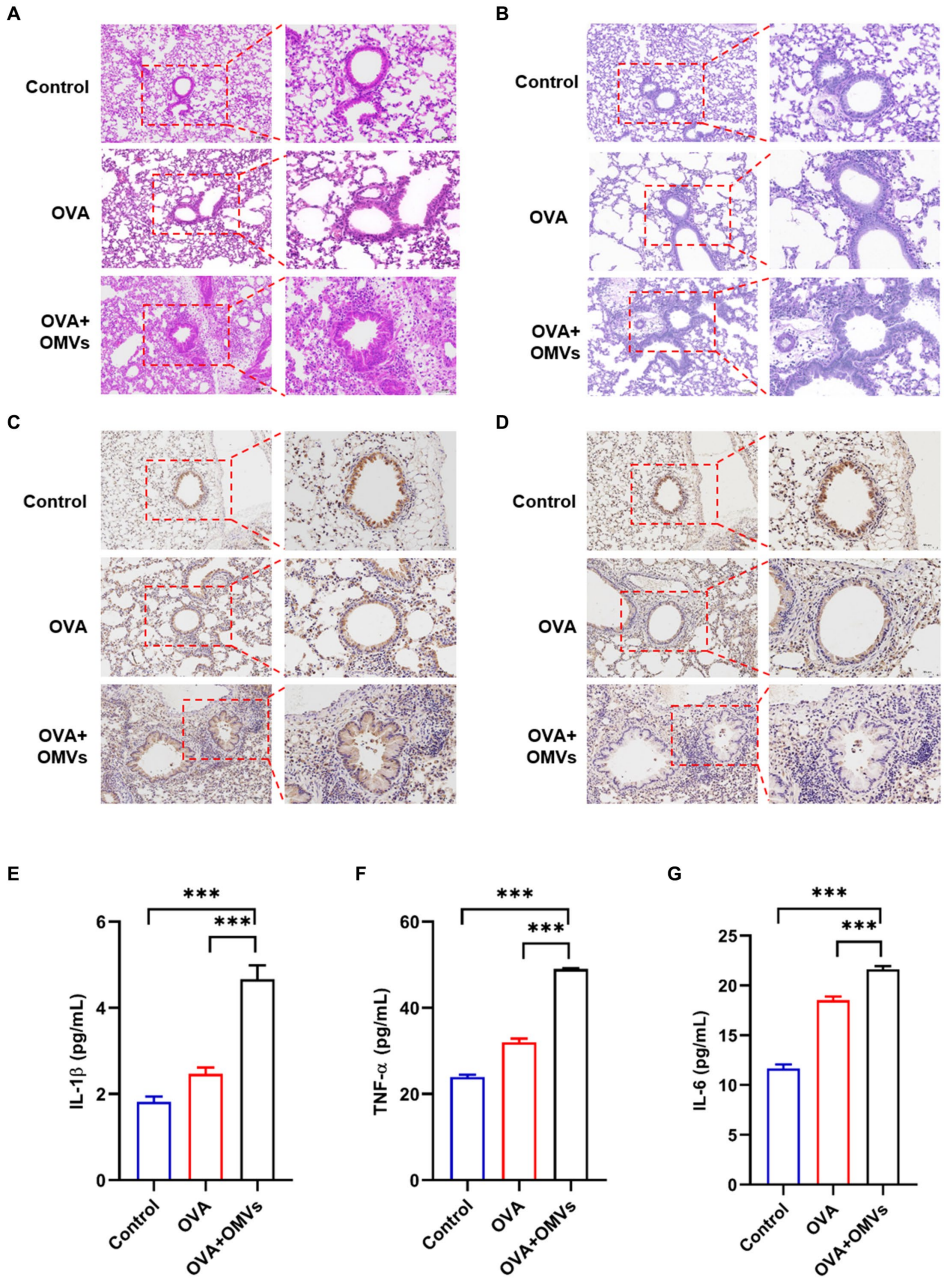
Inhalation of NTHi OMVs increased the mice’s airway obstruction. **(A)** Representative images of H&E staining in control, OVA induced and NTHi OMVs combined with OVA-sensitized mice. Scale bar: left row 100 μm, right row 50 μm. **(B)** Representative images of PAS staining in control, OVA induced and NTHi OMVs combined with OVA-sensitized mice. Scale bar: left row 100 μm, right row 50 μm. **(C)** Representative immunohistochemistry stained of MUC5AC in control, OVA induced and NTHi OMVs combined with OVA-sensitized mice. Scale bar: left row 100 μm, right row 50 μm. **(D)** Representative immunohistochemistry stained of GRα in control, OVA induced and NTHi OMVs combined with OVA-sensitized mice. Scale bar: left row 100 μm, right row 50 μm. **(E)** *IL*-1β levels of BALF detected in control, OVA induced and NTHi OMVs combined with OVA-sensitized mice by Elisa assay (*n* = 3). **(F)** TNF-α levels of BALF detected in control, OVA induced and NTHi OMVs combined with OVA-sensitized mice by ELISA assay (*n* = 3). **(G)** IL-6 levels of BALF detected in control, OVA induced and NTHi OMVs combined with OVA-sensitized mice by Elisa assay (*n* = 3). Each group contained 5 mice at least and each ELISA assay were conducted three times independently (*p < 0.05, ***p < 0.001).

To confirm the inflammation caused by NTHi OMVs inhalation in OVA-induced mice, we conducted histological analyses of lung tissues using Hematoxylin and Eosin (H&E) and immunohistochemical staining assays. Compared to the phosphate-buffered saline (PBS) group, OVA+NTHi OMVs significantly disrupted the morphology of airway epithelial cells, with thickened and narrowed lumens and more inflammatory cell infiltration around bronchi (Figure 2A; Supplementary Figure S1A). Periodic Acid-Schiff (PAS) staining showed a significant increase in the number of airway epithelial cupping cells compared to the control group (Figure 2B; Supplementary Figure S1B). Immunohistochemistry staining of MUC5AC was performed to confirm the mucus secretory capacity and revealed that the intensity of MUC5AC was significantly higher in OVA+NTHi OMVs mice than in the control group (Figure 2C; Supplementary Figure S1C). To further assess the hormonal sensitivity of the bronchi, we examined immunohistochemistry staining of GRα. Representative images showed that the intensity of GRα was weaker in OVA+NTHi OMVs mice than in the control group, suggesting that sensitivity to glucocorticoids was downregulated in OVA+NTHi OMVs mice (Figure 2D; Supplementary Figure S1D). The IHC scores from the stained images were statistically analyzed in Supplementary data (Supplementary Figure S1).

These results suggest that OVA combined with NTHi OMVs-induced airway obstruction through epithelial injury, mucus hypersecretion, and poor glucocorticoid response. In addition, we assessed the levels of inflammatory cytokines, such as IL-1β, tumor necrosis factor (TNF)-α, and IL-6, in bronchoalveolar lavage fluid (BALF) using enzyme-linked immunosorbent assay (ELISA) assays. The levels of IL-1β, TNF-α, and IL-6 in OVA-sensitized mice were upregulated compared to the control group, and NTHi OMVs inhalation further aggravated the release of these inflammatory cytokines (Figures 2E–G). These findings suggest that inhalation of NTHi OMVs in OVA-sensitized mice induces and exacerbates inflammation infiltration in the airway.

### NTHi OMVs inhalation promoted neutrophilic inflammation in OVA-sensitized mice

**Figure 3:**
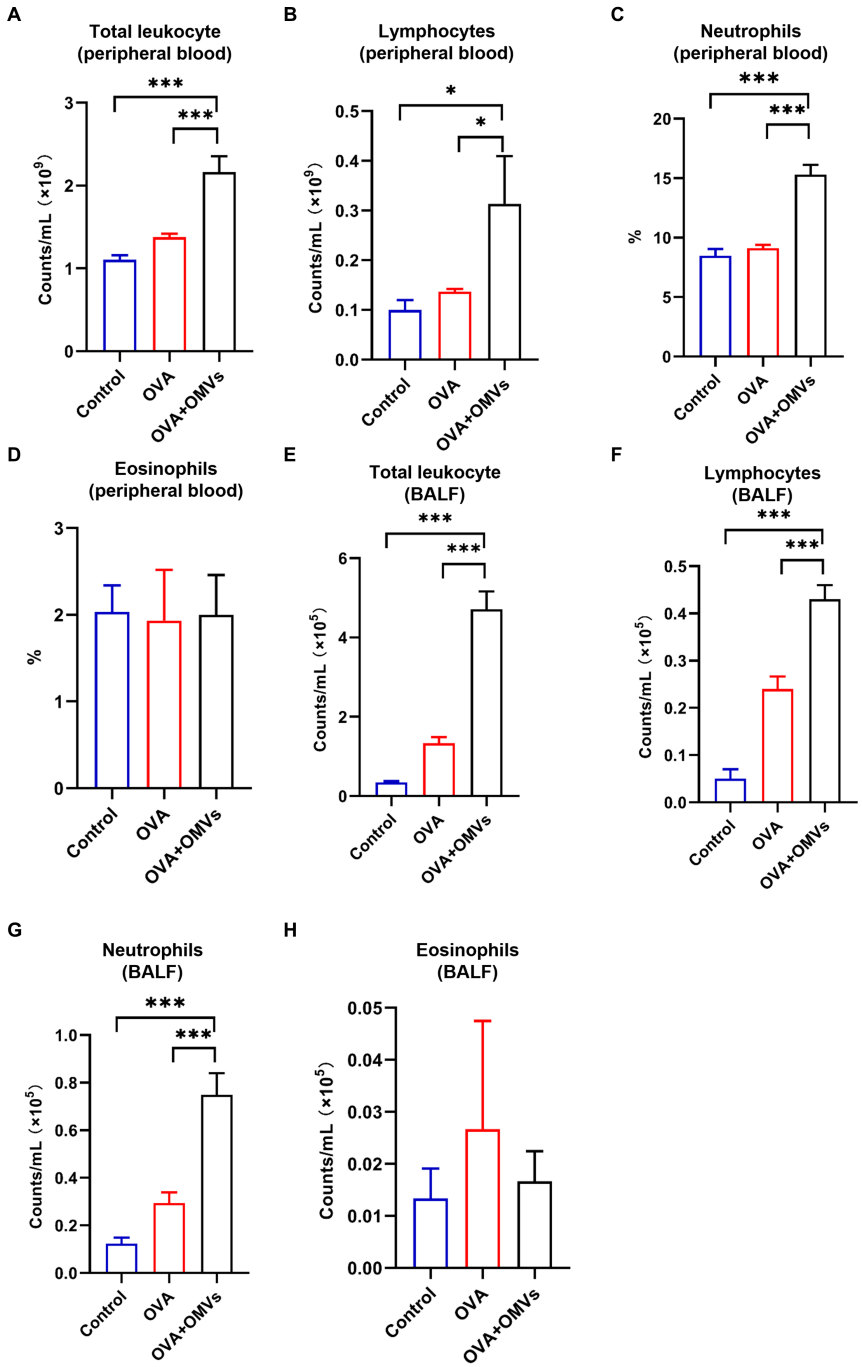
Neutrophilic inflammation induced by NTHi OMVs inhalation in OVA-sensitized mice. **(A)** Total counts of blood flow leukocytes checked by automatic blood cell counter in control, OVA induced and NTHi OMVs combined with OVA-sensitized mice. **(B)** Total lymphocytes from mice blood checked by automatic blood cell counter in control, OVA induced and NTHi OMVs combined with OVA-sensitized mice. **(C)** The percent of neutrophils in mice blood confirmed by automatic blood cell counter in control, OVA induced and NTHi OMVs combined with OVA-sensitized mice. **(D)** The eosinophils percent in mice blood confirmed by automatic blood cell counter in control, OVA induced and NTHi OMVs combined with OVA-sensitized mice. **(E)** Total BALF leukocytes counts checked by automatic blood cell counter in control, OVA induced and NTHi OMVs combined with OVA-sensitized mice. **(F)** Total lymphocytes from mice BALF checked by automatic blood cell counter in control, OVA induced and NTHi OMVs combined with OVA-sensitized mice. **(G)** The neutrophil counts in BALF checked by automatic blood cell counter in control, OVA induced and NTHi OMVs combined with OVA-sensitized mice. **(H)** The eosinophils percent of BALF examined from control, OVA, and NTHi OMVs +OVA groups. Each group contained 5 mice at least (*p < 0.05, ***p < 0.001).

Neutrophilic asthma is characterized by neutrophils accounting for more than 65% of total leukocytes in sputum, in contrast to eosinophils, which are less than 2.5% (Clementi and Murphy, 2011). To determine whether the OVA+NTHi OMVs-induced mice model exhibited typical indicators of neutrophilic asthma, we tested blood and bronchoalveolar lavage fluid (BALF) samples. Total leukocyte counts were increased in the blood (Figure 3A) and BALF (Figure 3E) of OVA+NTHi OMVs mice. At the same time, the counts and percentage of lymphocytes were correspondingly upregulated in BALF and blood (Figures 3B,F). Furthermore, the percentage of neutrophils in blood and BALF were increased compared to the control group and OVA group (Figures 3C,G). The counts and percentage of eosinophils in BALF were increased, but there was no significant statistical difference in BALF or blood (Figures 3D,H). These results suggest that NTHi OMVs inhalation in OVA-sensitized mice can induce neutrophilic asthma.

### NTHi OMVs inhalation activated the signaling pathways associated with inflammation and immune response in OVA-sensitized mice

**Figure 4:**
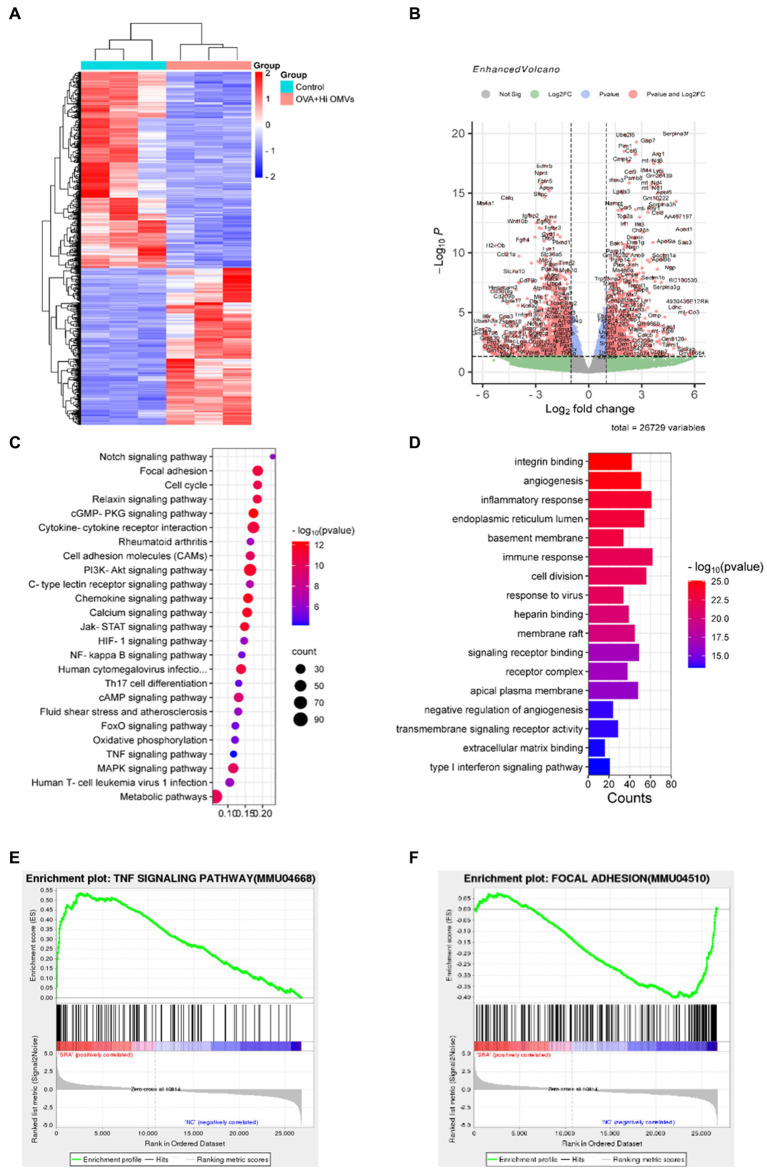
Activated signaling pathways in NTHi OMVs inhalation in OVA-sensitized mice. **(A)** Heat map clustered by differentially expressed genes in control and NTHi OMVs combined with OVA mice. **(B)** Volcano Plot distribution of differentially expressed genes in control and NTHi OMVs + combined with OVA mice. **(C)** GSEA analysis of involved signaling pathways. **(D)** KEGG signaling pathway enrichment analysis of differentially expressed genes. **(E)** TNF signaling pathway-related plots enrichment clustered in control and NTHi OMVs combined with OVA mice. **(F)** Focal adhesion signaling pathway-related plots enrichment clustered in control and NTHi OMVs combined with OVA mice.

To explore the mechanism of inhalation of NTHi OMVs inducing the pathogenesis of OVA-sensitized mice, we established a model to identify the differentially expressed genes and activated signaling pathways between the PBS and OVA+NTHi OMVs groups and explore the potential mechanisms of asthma pathogenesis induced by OVA+NTHi OMVs. We performed high-throughput sequencing analysis of lung tissue from the control and OVA+NTHi OMVs groups, using |log2 (FoldChange)| > = 1 and padj ≤ 0.05 to screen the differential genes. We identified 1,534 differentially expressed genes, including 681 upregulated and 853 downregulated genes in the OVA+NTHi OMVs group. These differentially expressed genes were clustered for heat mapping (Figure 4A), and a Volcano Plot showed each gene regulated by NTHi OMVs inhalation in OVA-sensitized mice (Figure 4B). Further gene set enrichment analysis (GSEA) analysis revealed that OVA+NTHi OMVs-induced significant upregulation of signaling pathways related to inflammatory response, such as the TNF signaling pathway and IL-17 signaling pathway, and a significant downregulation in cell adhesion molecules (CAMs) and focal adhesion (Figure 4C). We performed gene ontology (GO) enrichment analysis of these differential genes, which showed significant enrichment in the inflammatory response, immune response, and type I interferon signaling pathway, among other biological processes related to inflammation and immunity (Figure 4D).

These data suggest that intercellular adhesion and junctions were disrupted by OVA+NTHi OMVs treatment, which further increased the inflammatory response. We performed the Kyoto Encyclopedia of Genes and Genomes (KEGG) signaling pathway enrichment analysis of the differentially expressed genes to explore the altered signaling pathways in OVA+NTHi OMVs. The results showed that differentially expressed genes were enriched in the TNF signaling pathway and focal adhesion signaling pathways associated with inflammatory response (Figures 4E,F). These analyses suggest that NTHi OMVs induce a substantial activation of inflammation-related signaling pathways in OVA-sensitized mice.

### Inhalation of NTHi OMVs located in mouse epithelium cells

**Figure 5:**
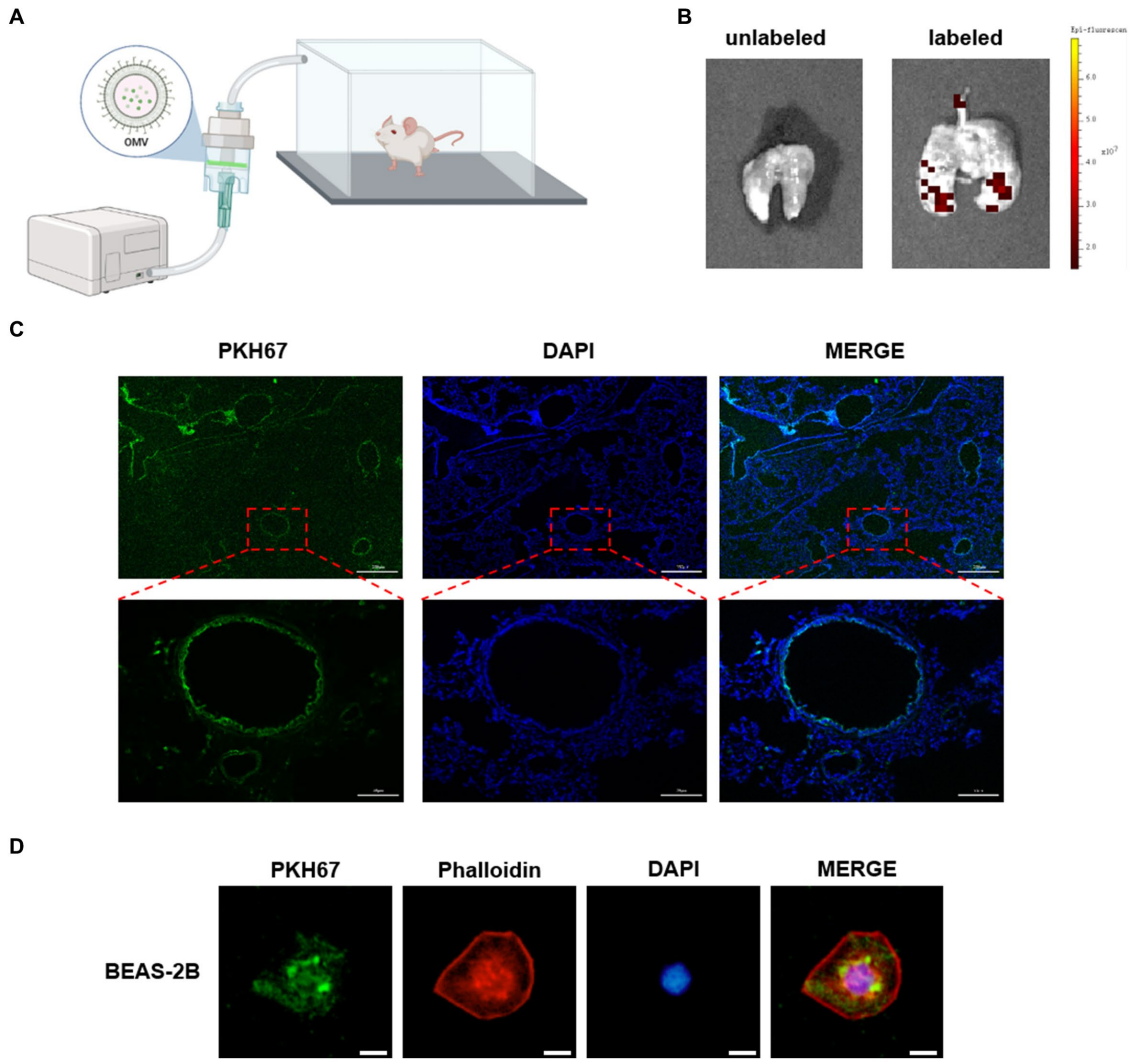
NTHi OMVs targeted airway epithelium. **(A)** The device designed for mice aerosol inhalation of NTHi OMVs in mice. **(B)** Representative images of NTHi OMVs aerosol inhalation by Animal Imaging Technology. **(C)** Representative images of NTHi OMVs inhalation absorbed by airway epithelium collected by lung tissue frozen section. Scale bar: top 250 μm and bottom 50 μm. **(D)** Representative images of NTHi OMVs colocalization with BEAS-2B cell by Immunofluorescence assays. Scale bar: 10 μm.

To identify the receptor cell of NTHi OMVs in the mouse airway, we administered PKH67-labeled OMVs to mice as an aerosol using a nebulizer device for 10 min, as illustrated in Figure 5A. Six hours later, the mice were killed by CO_2_ exposure, and lungs were removed and captured by fluorescence imaging. Representative images showed that PKH67-labeled OMVs were present in the lungs of mice treated with NTHi OMVs nebulizer, in contrast to the PBS treatment group (Figure 5B). This result suggests that NTHi OMVs were able to enter and settle in the upper and lower respiratory tracts. To further confirm the receptor cell of NTHi OMVs, we performed frozen sections of the mice’s lungs to observe the green fluorescence within the lung tissue. The green fluorescence was concentrated in the airway epithelium of the mice (Figure 5C; Supplementary Figure S3A). Additionally, we co-cultured PKH67-labeled OMVs with BEAS-2B cells for 6 h, and Phalloidin was used to stain BEAS-2B cells. Representative images of the co-location of OMVs and BEAS-2B cells are shown in Figure 5D. Our data indicate that aerosolized OMVs released from NTHi can be delivered to and adhere to the epithelium of the mouse respiratory tract.

### Upregulation of IL-1β in NTHi OMVs combined with OVA-sensitized mice

**Figure 6:**
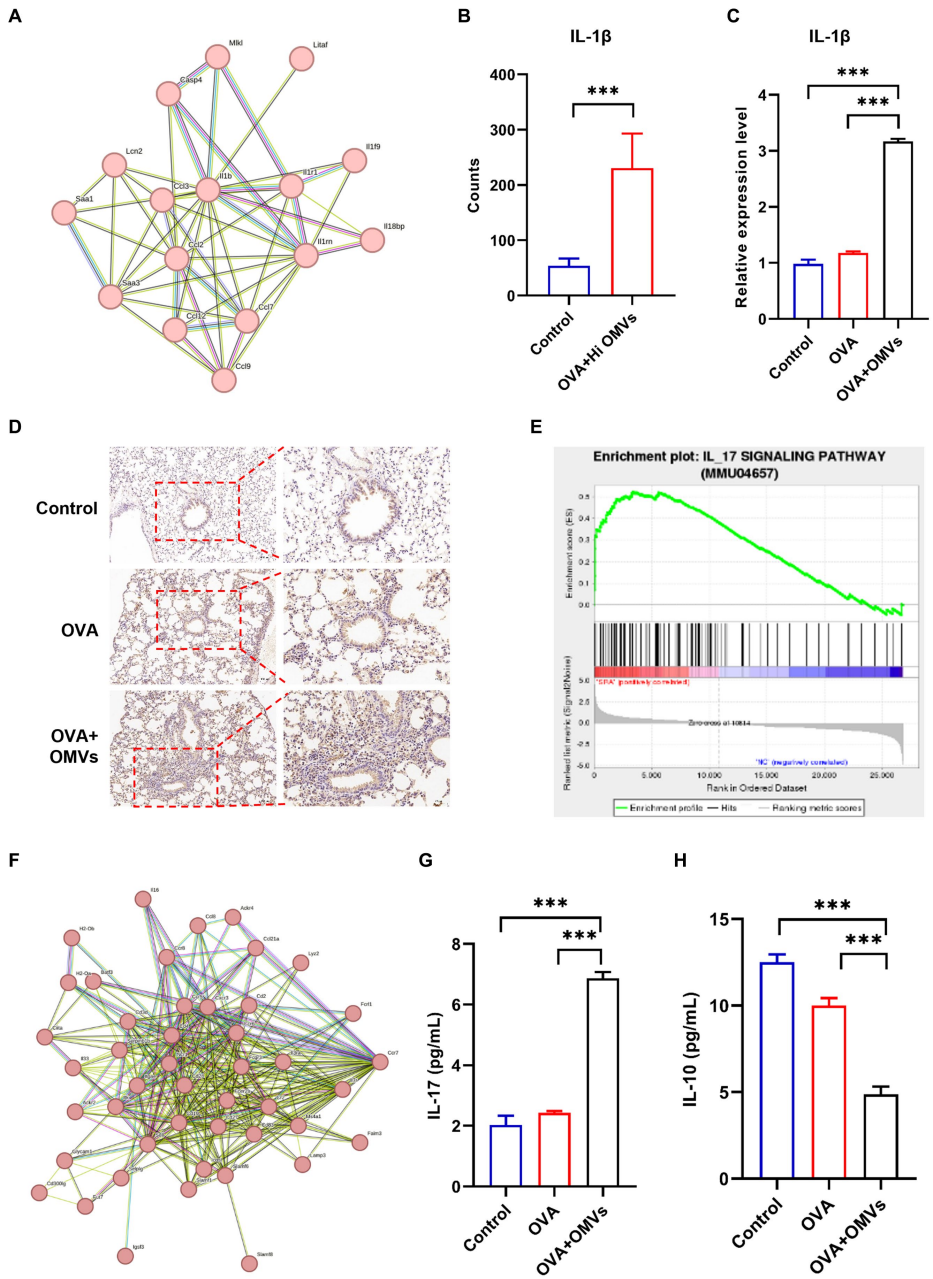
Upregulation of IL-1β in NTHi OMVs combined with OVA-sensitized mice. **(A)** IL-1β was enriched by the Search Tool for the Retrieval of Interacting Genes (STRING) database in NTHi OMVs-induced neutrophilic mice. **(B)** The mean counts of IL-1β in immune response in control and OVA combined with NTHI OMVs-induced neutrophilic mice by sequencing analysis. **(C)** IL-1β expression in mice lung tissue in control, OVA and OVA combined with NTHI OMVs-induced neutrophilic mice by qRT-PCR. **(D)** Combined with NTHI OMVs-induced neutrophilic mice by IHC assays. Scale bar: the left row 100 μm, right row 50 μm. **(E)** *IL*-17 signaling pathway enriched by sequencing analysis. **(F)** IL-17 enriched through STRING database. **(G)** IL-17 expression in BALF confirmed by ELISA assays in control, OVA, OVA combined with OMVs groups. **(H)** *IL*-10 expression in BALF confirmed by ELISA assays in control, OVA, OVA combined with OMVs groups. Each ELISA or qRT-PCR assays were conducted 3 times independently (*p < 0.05, ***p < 0.001).

The sequencing analysis showed that inflammation and immune response-related signaling pathways were significantly upregulated in NTHi OMVs-induced neutrophilic mice. We also found that IL-1β was enriched in several inflammatory response-related signaling pathways by the Search Tool for the Retrieval of Interacting Genes (STRING) database in NTHi OMVs-induced neutrophilic mice (Figure 6A), which may be an essential gene in the immune response of NTHi OMVs-induced neutrophilic mice. The levels of IL-1β were significantly upregulated, with the mean of counts increasing from 53.7 to 231.0 (Figure 6B).

**Figure 7:**
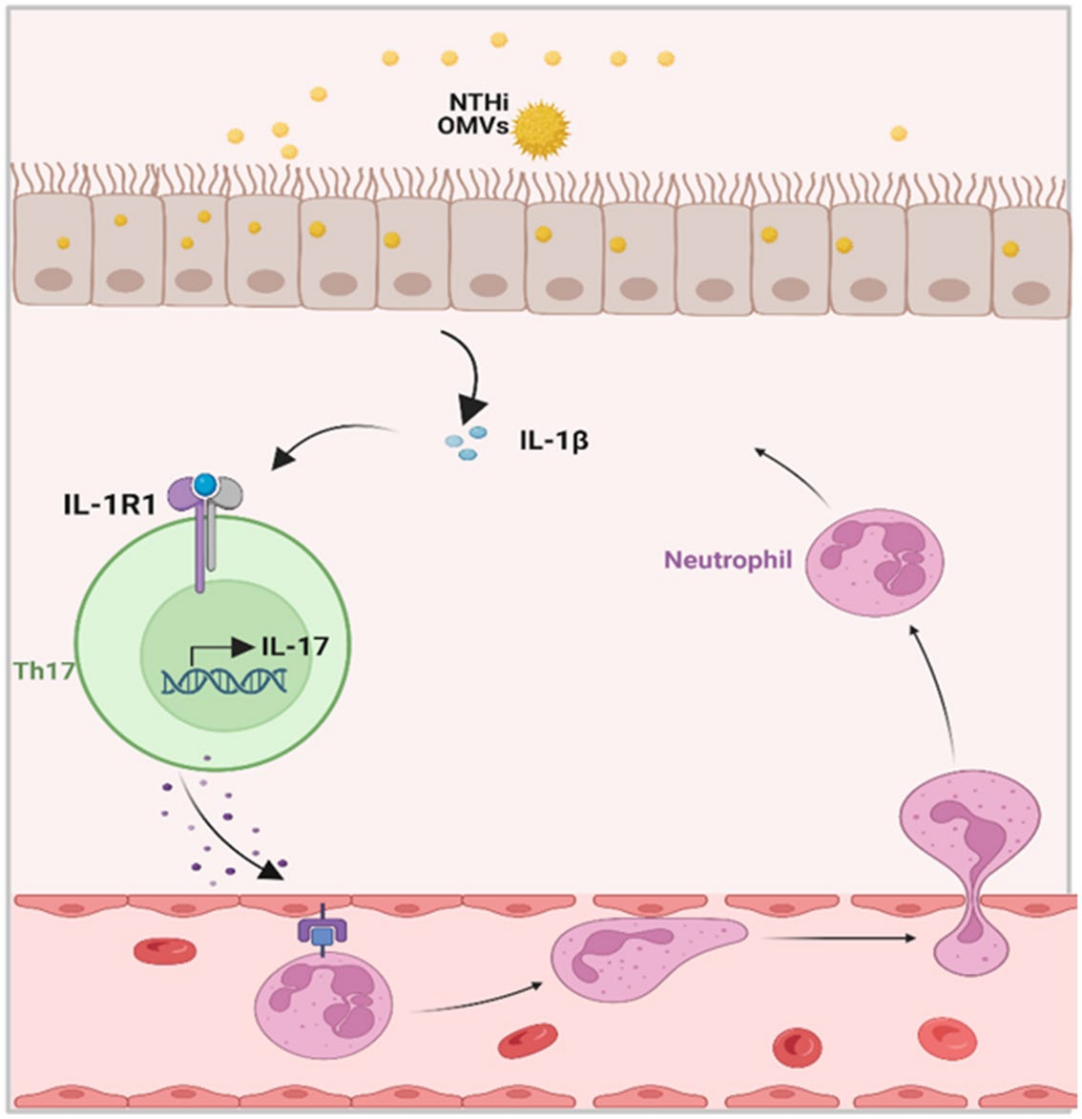
Schematic diagrams of NTHi OMVs contributed to the progress of neutrophilic asthma. OMVs released from NTHi transported into airway epithelium and released IL-1β. Furthermore, this would bind to IL-1R1 on Th17 cells and facilitate the IL-17 transcription. At last, IL-17 induced the neutrophil chemotaxis to neutrophilic inflammation in the airway.

We validated the transcriptomic sequencing data by RT-PCR, which showed that IL-1β expression was significantly upregulated in the lung tissue of the NTHi OMVs+OVA mice in contrast to the control and OVA group (Figure 6C). We also examined IL-1β expression levels in lungs by immunohistochemistry staining and found the most strong and intensive staining of IL-1β in NTHi OMVs+OVA mice (Figure 6D; Supplementary Figure S2A). The expression of IL-1β stained were calculated and showed in Supplementary Figure S2. Furthermore, th17 differentiation was enriched by sequence analysis, and we found that the IL-17 signaling pathway was involved in NTHi OMVs+OVA mice (Figure 6E). The STRING database was used to identify that IL-17 was enriched (Figure 6F). We also confirmed the IL-17 and IL-10 expression in BALF by ELISA assays, and the results showed that the expression of IL-17 was upregulated and IL-10 was downregulated in BALF of OMVs+OVA mice (Figures 6G,H). These results suggest that the transcriptional levels and secretion of IL-1β, IL-17 were upregulated and IL-10 was downregulated in the lung tissue of NTHi OMVs-induced neutrophilic asthma mice and may be involved in the immune response mediating the increased inflammatory response (Figure 7).

## Discussion

A previous study confirmed non-typeable *Haemophilus influenzae* combined with OVA-induced steroid-resistant asthma (SRA) in mice (Wang et al., 2019). In our study, we discovered that OMVs released from non-typeable *Haemophilus influenzae* could enter the lung tissue of mice *via* aerosolization and induce neutrophilic inflammation in OVA-sensitized mice. Through sequencing analysis, we observed that lung tissue from the NTHi OMVs group had significantly enriched inflammatory and immune-related signaling pathways, and the transcription and release of IL-1β and IL-17 were significantly increased. Our research identified the role of OMVs from non-typeable *Haemophilus influenzae* in the pathogenesis of neutrophilic asthma, with IL-1β potentially playing an essential role in this process.

At present, most studies use egg albumin (OVA) as allergen and aluminum hydroxide (alum) as adjuvant for the construction of asthma model, which has been a classic method in asthma research (Corazza and Kaufmann, 2012). For example, OVA and Alum were injected on days 0 and 7 and the OVA challenge, which is the construction of an eosinophile-dominated asthma model, was performed on days 14 and 22 (Casaro et al., 2019). In order to explore the effect of NTHi OMVs in our experiment, we did not refer to this method. Firstly, we did not use Alum, and secondly, our OVA processing time was shortened, so the OVA group alone did not cause severe asthma-related symptoms and reactions in mice. The addition of NTHi OMVs resulted in an increased asthma response in the mice.

Transcriptomic analysis of airway samples from NA patients showed that IL-17 induced chemokines (CXCL1, CXCL2, CXCL3, and CSF-3) and neutrophil chemokines (IL-8) were significantly increased (Wang et al., 2019), suggesting that the immune abnormality mediated by Th17/Treg immune imbalance is an important pathogenesis of NA. Previous studies have suggested that asthma is a chronic airway inflammatory disease induced by Th1/Th2 immune imbalance mainly mediated by Th2. With the deepening of research, it was found that the Th2 imbalance hypothesis could not fully explain the pathogenesis of all asthma patients, and Th17/Treg immune imbalance played a crucial role in airway inflammation and airway remodeling in asthma (Bisgaard et al., 2007). Th17 cells can secrete pro-inflammatory cytokines such as IL-17A, IL-17F and IL-22, which promote airway neutrophil infiltration, airway mucocyte metaplasia and airway remodeling. Treg cells inhibit inflammatory response by releasing anti-inflammatory factors IL-10 and TGF-β, promoting tolerance to allergens and preventing allergic diseases (Sarwer et al., 2015). Through high-throughput sequencing analysis of lung tissue of asthmatic mice sensitized by NTHi outer membrane vesicles combined with OVA, we found that NTHi OMVs promoted significantly up-regulation of IL-17 signaling pathway in lung tissue of asthmatic mice induced by OVA. Further analysis showed that IL-1β was enriched in IL-17 signaling pathway, Th17 cell differentiation and TNF signaling pathway. Thus, NTHi-derived OMVs induce airway epithelial cells to release IL-1β, mediating Th17/Treg immune imbalance, leading to recruitment of neutrophils.

NTHi is a Gram-negative, conditional pathogenic bacteria in the respiratory tract and the most commonly isolated species in neutrophilic asthma (Taylor et al., 2018). NTHi promotes neutrophilic asthma by causing mucus hypersecretion, Th1/Th2, and Th17/Treg imbalances (Zhang et al., 2020). However, most studies have focused on NTHi thallus to understand the mechanisms of neutrophilic asthma, with few studies exploring the pathogenicity and pathogenesis of neutrophilic asthma using the components of NTHi. Our study observed high airway responsiveness, airway lumens narrowing, thickening of airways, and infiltration of inflammatory cells around the bronchi. Inflammatory cytokines were also released into the BALF in the NTHi OMVs + OVA group. Furthermore, the upregulation of neutrophil percent and lymphocytes in blood and BALF indicated the type of neutrophilic asthma induced by NTHi OMVs in OVA-sensitized mice. Although sequencing analysis indicated that the IL-17 signaling pathway was significantly activated, we did not analyze the difference in Th1/Th2/Th17/Treg distribution in each mice group, which was a limitation. These findings suggest that we have built a model of neutrophilic asthma, and IL-17 target therapy may be a promising candidate for neutrophilic SRA in the clinic.

A previous study by Sharpe et al. (2011) demonstrated that NTHi OMVs could be internalized by the human pharynx and primary chinchilla middle ear epithelial cells as immune effectors. In our study, we observed that vesicles from NTHi could be absorbed by bronchial epithelial cells and elicit the release of IL-1β in mice BALF. Further histological analyses described the typical pathological changes of neutrophilic asthma induced by NTHi OMVs. Airway epithelial cells act as a physical barrier between the body and the environment and as active immune cells (Hammad and Lambrecht, 2021). Recently, the latest research has established the central role of airway epithelial cells and cytokines in the pathogenesis of eosinophilic asthma (Corazza and Kaufmann, 2012). However, whether airway epithelial cells contribute to the development of neutrophilic asthma has not been explored. Our findings indicate that airway epithelial cells are the receptor cells that absorb NTHi-released OMVs and then release IL-1β, providing significant evidence to prove that airway epithelial cells and epithelial-derived cytokines contribute to neutrophilic asthma, as shown in Figure 7.

In our study, IL-1β was significantly upregulated in the lung tissue of the neutrophilic asthma group and significantly increased in BALF. Importantly, the release of IL-1β may be due to the release of inflammasomes related pathways in neutrophilic asthma (Rossios et al., 2018). Vanaja et al. (2016) reported that LPS from bacterial OMVs can mediate cytosolic localization of LPS and caspase 11 activation to defend against infection, which suggesting that the OMVs released from NTHi could transport LPS to the airway epithelium and activate LPS/caspase 11 signaling to release IL-1β. Therefore, further study should focus on NTHi OMVs in inducing cell pyroptosis to promote the pathogenesis of neutrophilic asthma in mice. Targeting the release of OMVs may be a useful therapy to control neutrophilic inflammation in neutrophilic asthma.

## Materials and methods

### NTHi culture and separation of OMVs

The NTHi strain was cultured overnight in sBHI broth medium at 37°C and 180 rpm. The culture was centrifuged at 10,000 × g for 10 min at 4°C to remove thalli precipitation. The resulting supernatant was enriched to one-sixth of the initial volume using a 100-kDa column (Millipore). The concentrated supernatant was filtered through a 0.22-μm filter (Millipore) to remove any bacteria further. The resulting supernatant was transferred to a thick-wall overspeed centrifuge tube and centrifuged at 100,000 × g for 1 h at 4°C. Following centrifugation, the supernatant was removed, leaving behind the OMVs precipitation.

### NTHi OMVs characterization

The sizes and concentrations of OMVs were examined using a NanoSight NS300 Nanoparticle Tracking Analysis (NTA) device (Malvern Instruments) equipped with a 488-nm blue laser, a 20× lens, and an EMCCD camera. 20 μg NTHi OMVs were diluted to a range of 1:1,000–1:100,000 in ultrapure PBS to minimize noise and analyzed at least three times, with the results being averaged. To label the OMVs, PKH67 (Merck) was added following the manufacturer’s protocol and imaged using fluorescence microscopy.

The purified precipitates were resuspended in 50 μL of 2.5% glutaraldehyde, and the fixed precipitated suspensions were added onto a copper net covered with a formvar membrane. The excess liquid at the edge of the copper net was absorbed using filter paper, and phosphotungstic acid with pH 6.5 was used for negative staining. After staining for 1 min, the dye was absorbed using filter paper, and ddH2O was added to wash away the excess negative dye solution on the copper net. The distilled water was then absorbed using filter paper, and the water was allowed to dry at room temperature before being observed using a transmission electron microscope at 80 kV. The size and shape of the purified OMVs were then observed.

### NTHi OMVs uptake in mouse lung tissue

Female BALB/c mice aged 6 weeks and weighing 18–20 g, which were specific-pathogen-free, were purchased from Beijing Charles River Laboratories Co., Ltd. After being adapted for 1 week, 50 μg PKH67-labeled NTHi OMVs were resuspended in 10 mL of 0.9% saline. The mice were placed in an airtight box shielded from light, and 10 mL of 0.9% saline with PKH67-labeled NTHi OMVs were atomized through a nebulizer. Excess carbon dioxide was injected into the mice 6 h later, and lung tissues were immediately taken for observation using INDEC BIOSYSTEMS (FluorVivo，INDEC, USA). Tissue slices were then frozen, and Mounting Medium with antifading (with DAPI) (Solarbio) was added to seal the slices. Fluorescence in the tissues was observed using fluorescence microscopy (Olympus Corporation).

### The NTHi OMVs infected mouse model

Following the NTHi-induced asthma mouse model (Essilfie et al., 2015), mice were exposed to atomized NTHi OMVs on day 10 and intraperitoneally injected with 50 μg of OVA (Sigma) in 200 μL of 0.9% saline on day 0. From days 12–15, mice in the OVA+NTHi OMVs and OVA groups were given 15 mL of 2% OVA aerosol inhalation, while mice in the control group were given an equal amount of 0.9% aerosol inhalation. On day 16, the resistance and compliance system (RC) small animal lung function apparatus (DSI Buxco) was used to detect airway resistance and dynamic lung compliance of mice in each group. Each group contained five mice.

### Determination of lung function in mice

The DSI Buxco RC FinePointe system (DSI Buxco) was used to test the airway resistance and dynamic compliance of mice to acetyl-β-methylcholine chloride (Sigma) at 0, 3.125, 6.25, 12.5, and 25 mg/mL. The mice were then given peritoneal anesthesia with 1% sodium pentobarbital. Once deeply anesthetized, the mice were fixed supine on an operating board, and the head was secured to maintain airway patency. Endotracheal intubation was performed after gas resection, and the surgical line was fixed bidirectionally. When the respiratory curve was stable, and no apparent spontaneous respiration was observed, the atomizing device was started, and the gradient concentration of acetyl-β-methylcholine chloride was activated successively. Airway resistance and dynamic lung compliance were then recorded in real time. Each group contained 5 mice at least.

### Histopathology evaluation

For histopathological evaluation, lung tissue was collected, fixed with paraformaldehyde, and embedded in paraffin. Then, 5-μm-thick sections were cut and stained with hematoxylin–eosin staining (H&E), PAS. The levels of related proteins were evaluated by using immunohistochemical staining as previously described (Song et al., 2018). The optical densities of the images were measured by Image ProPlus 6.0 (Media Cybernetics).

### RNA quantification and illumina sequencing

The total amount and integrity of RNA were assessed using the RNA Nano 6,000 Assay Kit of the Bioanalyzer 2100 system (Agilent Technologies). Sequencing was performed on the Illumina NovaSeq 6000, generating an end reading of 150-bp pairing. Sequencing by Synthesis is the basic principle of sequencing, whereby four fluorescent-labeled dNTPs, DNA polymerase, and splice primers were added to the sequenced flow cell and amplified. Each labeled dNTP releases the corresponding fluorescence as the sequence cluster extends the complementary chain. The sequencer captures the fluorescence signal, and computer software converts the optical signal into the sequencing peak to obtain sequence information of the fragment being tested. Novogene Co., Ltd. analyzed all data. The RNA-seq data were deposited in the Gene Expression Omnibus (GEO) database with accession number GSE222546.

### qRT-PCR

Quantitative real-time PCR (qRT-PCR) assay was conducted by Applied Biosystem 7,500 with SYBR (BL705A, Biosharp). Relative gene expression was calculated by using the 2^-ΔΔCT^ method. The primers of IL-1β used for qRT-PCR were ATGATGGCTTATTACAGTGGCAA as forward primer and GTCGGAGATTCGTAGCTGGA as reverse primer.

### Statistical analysis

ANOVA analysis was used for comparison of differences among multiple groups, and student t-test was used for comparison of differences between two groups. All statistical analyses were performed with SPSS23.0 software. Significant differences between groups were considered when the *p*-value was less than 0.05.

## Data availability statement

The datasets presented in this study can be found at the following link: https://www.ncbi.nlm.nih.gov/geo/query/acc.cgi?, Accession number: GSE222546.

## Ethics statement

The animal study was reviewed and approved by Animal Ethics Committee of the Changchun Wish Technology Company.

## Author contributions

YiZ: data analysis and writing. YL: formal analysis and validation. HW, YaZ, and PZ revised the manuscript. All authors contributed to the article and approved the submitted version.

## Funding

This work was supported by grants from the Natural Science Foundation of Jilin Province (No. 2017J037) (No. YDZJ202201ZYTS008 and YDZJ202201ZYTS528), Health Commission of Jilin Province (No. 2017J037), and the International Cooperation Project of Science and Technology Department of Jilin Province (No. 20210402012GH).

## Conflict of interest

The authors declare that the research was conducted in the absence of any commercial or financial relationships that could be construed as a potential conflict of interest.

## Publisher’s note

All claims expressed in this article are solely those of the authors and do not necessarily represent those of their affiliated organizations, or those of the publisher, the editors and the reviewers. Any product that may be evaluated in this article, or claim that may be made by its manufacturer, is not guaranteed or endorsed by the publisher.
