# Supplementary figures and images for: Aerosolization inhalation of non-typeable Haemophilus influenzae outer membrane vesicles contributing to neutrophilic asthma
Source: Front Microbiol. 2023 Jul 26;14:1226633. doi: 10.3389/fmicb.2023.1226633 (PMC10411346; doi:10.3389/fmicb.2023.1226633)

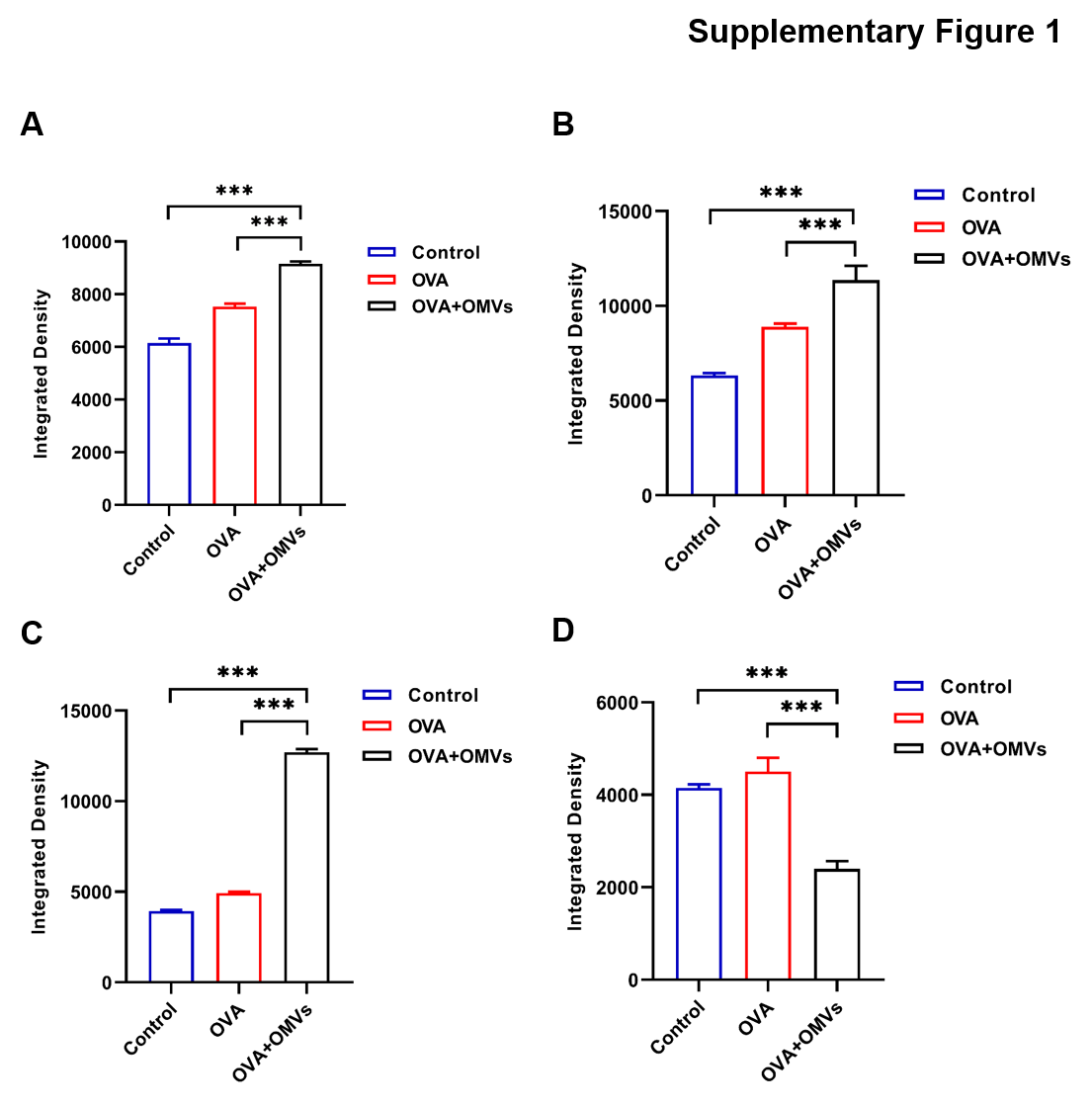

Supplement: Supplementary file 1 [file Image_1.tif]

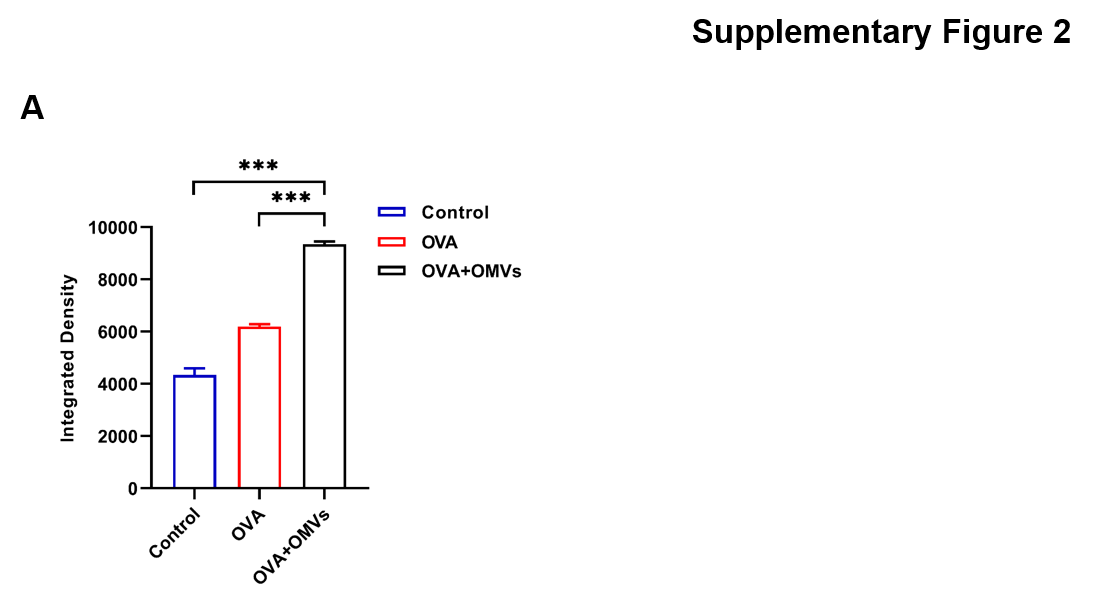

Supplement: Supplementary file 2 [file Image_2.tif]

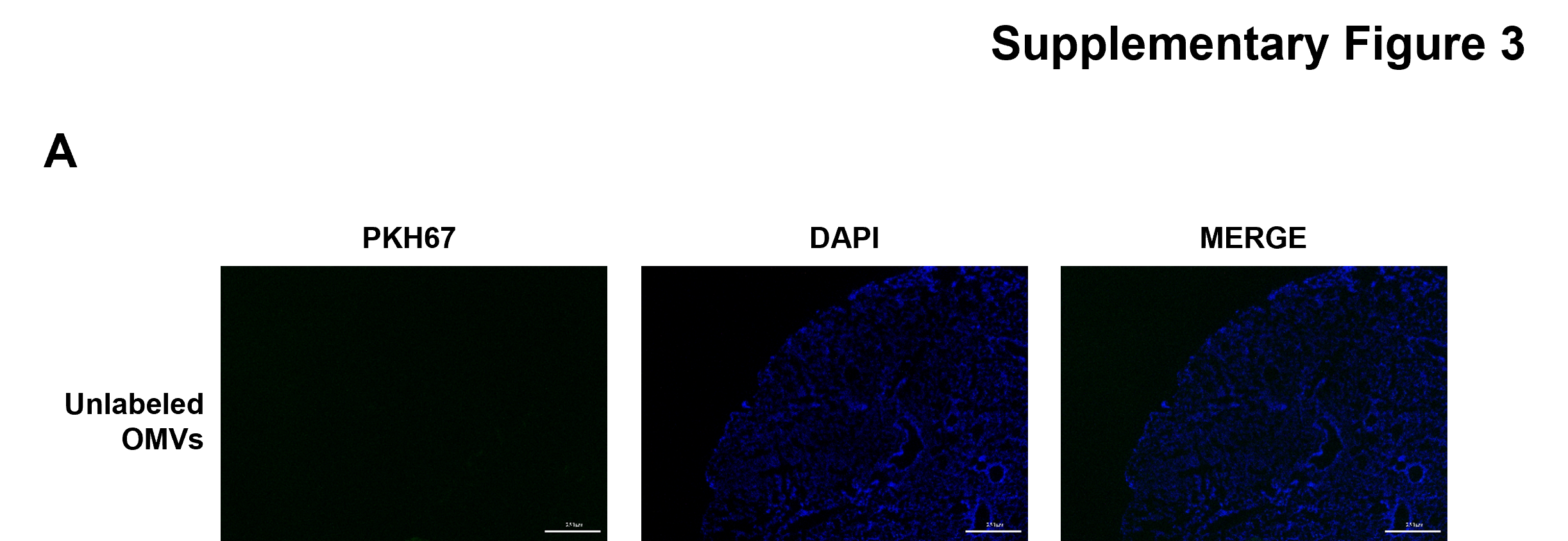

Supplement: Supplementary file 3 [file Image_3.tif]
